# Supplementary material for: TOR1AIP1-Associated Nuclear Envelopathies
Source: Int J Mol Sci. 2023 Apr 7;24(8):6911. doi: 10.3390/ijms24086911 (PMC10138496; doi:10.3390/ijms24086911)
Supplement: Supplementary file 1 [file ijms-24-06911-s001.zip › ijms-2247079-supplementary.pdf]

TOR1AIP1-ASSOCIATED NUCLEAR ENVELOPATHIES

Supplementary material

1. List of keywords and databases

Main concept	Keywords - Pubmed	Keywords - Ovid	Keywords - Scopus
LAP 1	• "LAP1”<br>• "LAP-1"<br>• "lamina associated polypeptide 1<br>• "LAP1A"<br>• "LAP1B"<br>• "LAP1C"<br>• "lamina-associated polypeptid*"<br>• "lamina-associated prot*"<br>• "TOR1AIP1"<br>• "Torsin 1A Interacting Protein 1"<br>• "Torsin-1A-Interacting Protein 1")	• LAP1.ti,ab,kf.<br>• LAP-1.ti,ab,kf.<br>• “lamina associated polypeptide 1”.ti,ab,kf.<br>• lamina-associated polypeptid*.ti,ab,kf.<br>• lamina-associated prot*.ti,ab,kf.<br>• (lamina adj3 associated adj3 (prot* or polypept*)).ti,ab,kf.<br>• LAP1C.ti,ab,kf.<br>• LAP1B.ti,ab,kf<br>• LAP1A.ti,ab,kf.<br>• (LAP1 adj3 ('A' or 'B' or 'C')).ti,ab,kf.<br>• TOR1AIP1.ti,ab,kf<br>• “Torsin 1A Interacting Protein 1”.ti,ab,kf<br>• (torsin adj3 interacting adj3 prot*).ti,ab,kf.<br>• torsin adj3 protein.ti,ab,kf<br>• TOR-1AIP1.ti,ab,kf<br>• (Torsi ajd3 1A ajd3 interacting ajd3 Protein ajd3 1).ti,ab,kf	• “Torsin-1A-Interacting Protein 1"<br>• “Torsin 1A Interacting Protein 1"<br>• “LAP1”<br>• “ LAP-1”<br>• lamina associated polypeptide 1<br>• lamina-associated polypeptid*<br>• lamina-associated prot*<br>• LAP1A<br>• LAP1B<br>• LAP1C<br>• TOR1AIP1<br>• lap1 W/3 (a OR b OR c)<br>• (torsi* W/3 (1a OR 1 OR a) W/3 interacting W/3 protein)<br>• (lamina W/3 associated W/3 (polypeptid* OR protein* OR peptid*))
Molecular basis/physiopathology of LAP1	**Structure**<br>• ("nuclear envelop*"<br>• "nuclear lamina"<br>• "nuclear membran*"<br>• " inner nuclear membran*"<br>• “nucleus”<br>• "INM"<br>• "Emerin"<br>• "Torsin"<br>• "TorsinA"<br>• "laminin*"<br>• “mutation*”<br>• "endoplasmic reticulum"<br>• "membrane protein*"<br>• “Nuclear protein”<br>• “Molecular”<br>• “structure”<br><br>**Physiopathology**<br>• "patholog*"<br>• "mutation*"<br>• "mechanism*”	**Structure**<br>• “nuclear lamina”.ti,ab,kf<br>• “nuclear envelop*”.ti,abkf.<br>• “nuclear membrane*”.ti,ab,kf.<br>• “inner nuclear membran*”.ti,ab,kf<br>• "INM".ti,ab,kf.<br>• nuclear envelope/ or nuclear lamina/<br>• (inner ajd3 nuclear ajd3 membran*).ti,ab,kf.<br>• "Emerin".ti,ab,kf.<br>• "TorsinA".ti,ab,kf<br>• (torsin adj3 "A").ti,ab,kf.<br>• laminin*.ti,ab,kf.<br>• Laminin/<br>• membrane proteins/ or nuclear proteins/<br>• Structure.ti,ab,kf.<br>• Molecular.ti,ab,kf.<br>• Molecular Structure/<br><br>**Physiopathology**<br>• Mutation*.ti,ab,kf.<br>• Mutation/<br>• patholog*.ti,ab,kf.<br>• Pathology, Molecular/<br>• mechanism*.ti,ab,kf.	**Structure**<br>• "nuclear membran*"<br>• "nuclear lamina"<br>• “Nuclear envelop*”<br>• “Inner nuclear membrane”<br>• (inner W/3 nuclear W/3 membran*)<br>• "INM"<br>• "Emerin"<br>• "Torsin"<br>• "TorsinA"<br>• (torsin W/3 "a")<br>• "laminin*"<br>• "endoplasmic reticulum"<br>• "Molecular"<br>• “Structure”<br><br>**Physiopathology**<br>• "Pathology”<br>• “mutation*”<br>• “patholog*”<br>• ‘mechanism*”

Clinical spectrum of LAP1-related diseases	**Health and diseases**<br>• "disease*"<br>• "Health"<br><br>**Muscular**<br>• myopath*<br>• "muscular dystroph"<br>• "Muscular dis*"<br>• "LGMD2Y"<br>• "congenital myasthenic syndrom*"<br>• "CMS"<br><br>**Cardiomyopathy**<br>• "cardiomyopath*"<br><br>**Movement Disorders**<br>• "dystoni* "<br>• "DYT1"<br><br>**Liver disease**<br>• "hepatic steatos*" OR NAFLD OR NASH OR "lipid droplet*" OR VLDL OR apoB OR "envelopath*")<br><br>Multisystemic disorders<br>• multisystem*<br><br>**Nucleopathies / envelopathies**<br>• "envelopath*"<br>• "nucleopath*"<br><br>**Deafness**<br>• "Deafness"<br>• "surdity"<br><br>**Cerebellar**<br>• cerebell*	**Health and diseases**<br>• Disease/<br>• Health/<br>• Health.ti,ab,kf.<br>• diseas*.ti,ab,kf.<br><br>**Muscular**<br>• myopath*.ti,ab,kf.<br>• muscular dystroph*.ti,ab,kf.<br>• (muscl* adj3 (dystro* or dis**)).ti,ab,kf.<br>• LGMD2Y.ti,ab,kf.<br>• (congenital adj3 myastheni*).ti,ab,kf<br>• cms.ti,ab,kf.<br>• muscular diseases/ or muscular dystrophies/ or myopathies, structural, congenital/<br><br>**Cardiomyopathy**<br>• cardiomyopath*.ti,ab,kf.<br>• Cardiomyopathies/<br><br>**Movement Disorders**<br>• dystoni*.ti,ab,kf.<br>• Dystonic Disorders/<br>• Dystonia/<br><br>**Liver diseases**<br>• Liver Diseases/<br>• "Liver diseas*".ti, ab, kf<br>• "Liver ajd3 diseas*".ti, ab, kf<br>• hepatic steatos*.ti,ab,kf.<br>• NAFLD.ti,ab,kf.<br>• NASH.ti,ab,kf.<br>• Non-alcoholic Fatty Liver Disease/<br>• apoB.ti,ab,kf.<br>• Apolipoproteins B/<br>• (apo adj3 'B').ti,ab,kf.<br>• VLDL.ti,ab,kf.<br>• lipoproteins, vldl/ or cholesterol, vldl/<br>• lipid droplet*.ti,ab,kf.<br>• Lipid Droplets/<br><br>**Multisystemic disorders**<br>• multisystem*.ti,ab,kf.<br>• "multisystem* ajd/2 (disorder* or diseas*)<br><br>**Nucleopathies / envelopathies**<br>• envelopath*.ti,ab,kf<br>• nucleopath*.ti,ab,kf	**Health and diseases**<br>• "disease*"<br>• "Health"<br><br>**Muscular**<br>• Muscular W/3 disease*<br>• "myopath*"<br>• muscular W/3 dystroph*<br>• "LGMD2Y"<br>• "congenital myasthenic syndrom*"<br>• congenital W/3 myastheni*<br>• "CMS"<br><br>**Cardiomyopathy**<br>• "cardiomyopath*"<br><br>**Movements disorders**<br>• "DYT1"<br>• "dystoni*"<br><br>**Liver diseases**<br>• "Liver W/2 diseas*<br>• "Liver diseas*"<br>• "hepatic steatos*"<br>• "NASH"<br>• "NAFLD"<br>• "non-alcoholic fatty liver disease*"<br>• apoB"<br>• "apolipoproteins b"<br>• apo W/3 "b"<br>• VLDL<br>• very low-density lipoproteins<br>• "lipid droplet*"<br><br>**Multisystemic disroder**<br>• "multisystem*"<br>• "multisystem* w/2 (disorder* or diseas*)<br><br>**Nucleopathies / envelopathies**<br>• "envelopath*"<br>• "nucleopath*"<br><br>**Deafness**<br>• "Deafness"<br>• "surdity"
